# Can smartwatches predict migraines? *Using machine learning (ML) with wearable-derived nocturnal autonomic nervous system (ANS) and sleep metrics for headache prediction*

**DOI:** 10.21203/rs.3.rs-8195467/v1

**Published:** 2025-12-01

**Authors:** Lewis E Tomalin, Benjamin R Kummer, Maya C Campbell, Asala N Erekat, Fred Cohen, Jessica Robinson-Papp, Bridget R Mueller

**Affiliations:** Icahn School of Medicine at Mount Sinai

**Keywords:** artificial intelligence, autonomic nervous system, electrodermal activity, heart rate variability, sleep

## Abstract

**Objective:**

To investigate whether nocturnal autonomic nervous system (ANS) activity and sleep metrics, as measured by a wearable device, can predict the occurrence of next-day migraine in patients with episodic and chronic migraine.

**Background:**

The unpredictable nature of migraine episodes contributes to disease burden and limits effective application of tailored preventive strategies. Small-molecule calcitonin-gene-related peptide (CGRP) receptor antagonists, available in limited monthly quantities, are now used for both acute and preventative treatment of migraine. Consequently, improving the ability to identify days with heightened migraine risk could significantly improve migraine management and treatment outcomes.

**Methods:**

In this prospective and observational study, adults with migraine (N = 10; 5 with chronic migraine and 5 with episodic migraine) wore the Empatica EmbracePlus^®^, smartwatch during sleep for a target duration of four weeks. Participants kept a headache diary recording days with no headache, non-migraine headache only, or migraine. First, group level analysis was performed using linear mixed-effects models (LMM). Next, personalized machine learning (ML) models were trained using nocturnal electrodermal activity (EDA), pulse rate variability (PRV), respiratory rate (RR), sleep duration, sleep interruptions, and awakenings to predict: (1) next-day migraine, and (2) next-day headache (both migraine and non-migraine). Performance was summarized using area under the receiver-operating and precision–recall curves (AUROC, AUPRC), sensitivity, specificity, accuracy, and precision. SHapley Additive exPlanation (SHAP) analyses identified the most influential predictors in highest performing next-day migraine and next-day headache models. Generalized Additive Models (GAM) explored nocturnal temporal dynamics of PRV and EDA.

**Results:**

Group level predictive performance assessed with LMMs did not reveal significant differences between ANS and sleep metrics on nights prior to no headache days, days with migraine, and days with non-migraine headache. However, individualized models using elastic-net regression, random forests, and gradient boosting machines showed modestly better-than-random AUROCs for next-day migraine prediction in 5/10 participants and next-day headache in 3/10 participants. For next-day migraine prediction models, four of five patients with episodic migraine showed better-than-random AUROCs; no patients with chronic migraine had better-than-random AUROCs. The highest-performing individualized models achieved moderate-to-good performance (AUROC 0.68 for next-day migraine and 0.81 for next-day headache). In highest performing models, SHAP analyses demonstrated sleep duration and a higher minimum PRV influenced next-day migraine and next-day headache probability, while EDA influenced next-day migraine, but not next-day headache. GAM analyses demonstrated that the first three hours after sleep onset and prior to awakening were time periods when PRV and EDA differed prior to a day with migraine or headache in these high performing models.

**Conclusions:**

Our findings indicate that applying individualized ML models to wearable-derived autonomic and sleep data may assist in the identification of heightened migraine risk and identified EDA, PRV, and sleep duration as important forecasting features. Our results provide a rationale for future studies that investigate how targeted medication and behavioral interventions on high-risk days may enhance therapeutic precision of migraine treatment and emphasize the importance of defining mechanistic subgroups of patients with migraine most likely to benefit from predictive modeling.

## INTRODUCTION

Migraine is a prevalent, debilitating, and economically burdensome neurological disorder affecting over 40 million Americans. [1, 2]. Small-molecule calcitonin-gene-related peptide (CGRP) receptor antagonists, known as gepants, are available in limited monthly quantities for the acute and preventative treatment of migraine. Recent evidence has demonstrated their efficacy in alleviating migraine prodrome symptoms [3], and ongoing clinical trials are evaluating targeted prophylactic use to prevent migraine during the high-risk premenstrual period [4, 5]. Therefore, the ability to accurately predict migraine has gained new clinical significance. Recent advancements in wearable biosensor technologies, along with machine learning (ML) methods, present unprecedented possibilities for continuous physiological monitoring and predictive modeling of migraine attacks [6].

It is well-established that changes in autonomic nervous system (ANS) activity occur during a migraine, including the premonitory phase [7–9]. The majority of evidence indicates there is sympathetic nervous system (SNS) predominance during a migraine attack, which is influenced by the severity of migraine pain [10]. Further, it has been demonstrated that in patients with morning migraines, norepinephrine levels are elevated several hours prior to awakening, indicating that alterations in the ANS may occur prior to the onset of pain [11]. Thus, we hypothesized that a wearable sensor with direct SNS monitoring capabilities may have the ability to forecast migraine episodes in quasi-real time, providing patients with clinically relevant information upon awakening regarding likelihood of experiencing a migraine later that day [12, 13].

Beyond predicting attacks, wearable technology has the potential to provide important insight into the pathophysiology of migraine and advance personalized medicine treatment approaches. While it is well-established that stress response systems including the SNS, are involved in migraine pathophysiology, challenges in the measurement of the SNS, as well as its context dependent activity, have impeded our understanding of this relationship [14]. For example, while stress increases vulnerability to migraine for some individuals, others report that it is a reduction in stress (or “let down”) that leads to migraine [15, 16]. Thus, continuous measurement of the ANS through wearable technology may provide important insight into the physiology that precedes migraine onset for an individual. In addition, insufficient sleep or reduced quality is known to be a powerful driver of migraine [17] and, thus, the ability to monitor ANS activity and sleep simultaneously may provide important insight into how sleep is altered prior to migraine episodes. Finally, wearable technologies may assist in identifying the most appropriate pharmacologic (e.g., beta blocker) and non-pharmacologic (e.g., sleep behavior modification) migraine treatments for an individual, facilitating the development of both holistic and personalized migraine treatment strategies [18, 19].

Our study has three main objectives. First, we aimed to determine the feasibility of having patients with migraine wear the commercial-grade, wrist-worn Empatica EmbracePlus^®^ (Empatica Inc., Cambridge MA) for a minimum of 4 weeks while keeping a headache diary. We chose the EmbracePlus over other wearables because it measures electrodermal activity (EDA) along with other more commonly used indices of ANS activity including pulse rate variability (PRV). In contrast to PRV, which reflects both sympathetic and parasympathetic influences, EDA reflects the activity of a purely sympathetic descending pathway that begins in the hypothalamus and ends with sympathetic efferents synapsing on eccrine glands [20, 21]. Our second objective was to develop person-specific predictive ML models using multimodal measurements of nocturnal ANS activity and sleep to forecast the occurrence of a next-day migraine. This person-specific approach acknowledges that migraine is heterogenous, and we did not expect that nocturnal sleep and ANS data would predict migraine reliably for every patient. Finally, given patients with migraine frequently report multiple headache types [22], we investigated if nocturnal ANS activity and sleep patterns preceding days with migraine differed from days with only non-migraine headache, and whether ANS activity and sleep patterns better predicted next-day migraine or next-day headache (migraine and non-migraine combined). We hope this study provides a foundation for the future study of wearables for migraine prediction and the development of personalized preemptive migraine management strategies.

## METHODS

### Study Population

Patients receiving care for migraine were prospectively identified at the David S. and Ruth L. Gottesman Center for Headache Treatment and Translational Research, Icahn School of Medicine at Mount Sinai (ISMMS), New York, between November 2023 and May 2025. Eligible participants were 18 years of age or older and met the International Classification of Headache Disorders, 3rd edition (ICHD-3) [23] criteria for migraine or chronic migraine, with or without aura, as determined by a headache specialist based on at least three months of headache diary data. Exclusion criteria included the presence of another headache or facial pain disorder, or a medical condition or treatment that would confound interpretation of wearable data (e.g., pacemaker, sleep-related disorder, or pregnancy). A study population of 10 was chosen to align with previous proof-of-concept studies evaluating feasibility of wearables [7, 24]. All study procedures were conducted in accordance with a protocol approved by the ISMMS Institutional Review Board, and written informed consent was obtained from all participants.

### Study Design

Following consent, participants received an EmbracePlus smartwatch, which was connected to the participants’ smartphone via Bluetooth and configured to stream real-time data into a secure, HIPAA compliant, cloud-based web dashboard (Empatica Health Monitoring Platform). The device required charging for one hour per day. Through the smartphone app, participants had real-time access to several ANS biomarkers including heart rate, skin temperature, and wear-time. We hypothesized nocturnal ANS and sleep activity may differ prior to a day with migraine versus non-migraine headache and distinguishing these headache types would improve model performance; thus, participants categorized each day as headache-free, a day with migraine, or a day with headache that did not meet migraine criteria using their preferred method of daily headache tracking [23].

#### Empatica EmbracePlus Data:

The Empatica EmbracePlus smartwatch collects the following raw data using a photoplethysmography sensor, EDA sensor, thermometer, accelerometer, and gyroscope: EDA (sampling frequency of 4 Hz), skin temperature (1 Hz), blood volume pulse (64 Hz), systolic peaks (64 Hz), and linear acceleration (64 Hz). Empatica also provides 1-minute aggregated data for skin temperature, sleep detection, body position, EDA, pulse rate variability (PRV), pulse rate (PR), respiratory rate (RR), and accelerometry data. Sleep metrics including time in bed (minutes), time asleep (minutes), time spent in bed awake, and time spent out of bed are also provided by Empatica based on ANS and actigraphy indices [25].

### Missing Data

A mixed-effects logistic regression analysis was conducted to examine the relationship between the probability of missing data for any physiological metric and reported headache day categorization. The analysis revealed no statistically significant association, indicating that the missingness was random. Missing values were therefore imputed using the Multivariate Imputation by Chained Equations (MICE) framework, with imputations performed separately for each participant and day to avoid bias related to headache day categorization (for additional information please see Supplemental Methods).

### Comparison of ANS and sleep features between nights preceding days with no pain, non-migraine headache, and migraine

Linear mixed-effects models (LMM) were used to compare nightly physiological measures between nights prior to no headache, migraine, and non-migraine headache. Each participant was modeled with a random intercept to account for multiple nights of data from the same individual. This structure adjusts for within-person correlation across repeated measurements and yields group-level estimates of physiological differences between nights. Statistical significance was set at a two-tailed p-value of 0.05 and corrected for repeated measures.

### Model Development

Individualized predictive models were built to estimate the likelihood of next-day headache or migraine based on nocturnal ANS and sleep data. The median, minimum, maximum, and standard deviation of 9 features including EDA, temperature, PR, PRV, RR, time in bed, time asleep, time awake in bed, and time spent out of bed were included in the models. Minimum, maximum, and standard deviation values were included to reflect the dynamic range and extremes of autonomic responses, categorizing stress reactivity more fully than averages alone. Features were derived from 5-minute intervals throughout each night. ANS activity during time spent out of bed (identified by actigraphy) were excluded from analysis to minimize the confounding effects of physical activity on ANS measurement.

Each 5-minute interval was treated as an independent observation contributing to that night’s overall physiological profile. The models first estimated the probability of next-day migraine/headache for each interval, and these interval-level probabilities are averaged to produce a single nightly prediction, representing the overall risk of next day migraine/headache. Three ML models: elastic-net regression, random forests, and gradient boosting machines, were evaluated for this individualized modeling framework [7]. These models were selected to capture a spectrum of modeling strategies: elastic-net regression for its interpretability and feature selection, random forests for handling complex nonlinearities and interactions, and gradient boosting for its strong predictive performance through sequential optimization.

#### Model Training and Performance Estimation:

**Figure 1:**
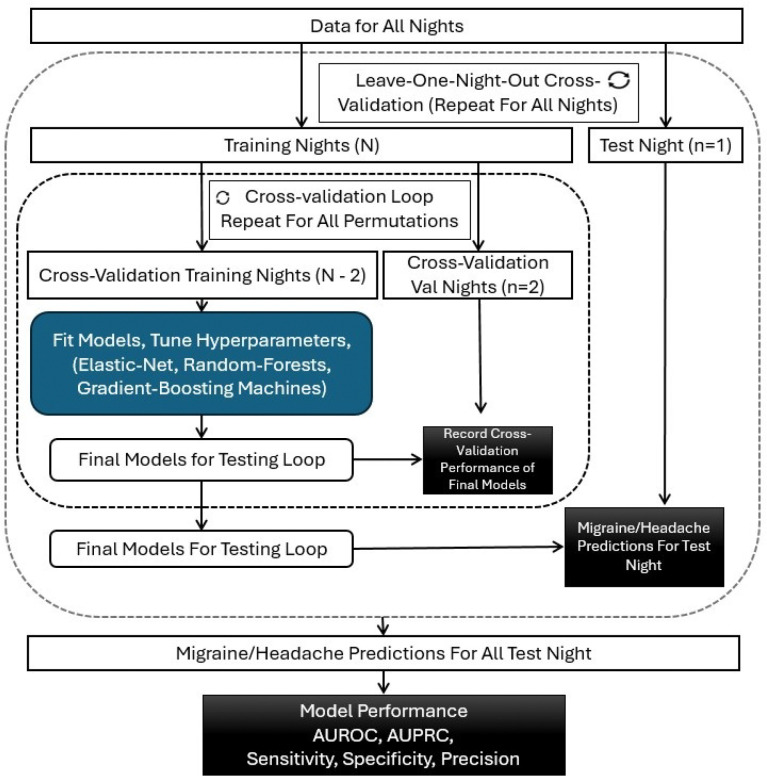
Schematic diagram of the machine learning strategy: Model performance was evaluated using a *leave-one-night-out*cross-validation approach. For each iteration, all data from a single night was held out as the test set, while the remaining nights served as the training set. This process was repeated so that each night was used once as the test set. Within each training set, hyperparameters were tuned using *leave-two-nights-out*cross-validation. Each validation fold consisted of one *headache* night and one *no-headache* night, and all possible such combinations were evaluated. The model with the optimal hyperparameters from the inner loop was retrained on the full training set and used to make predictions on the held-out test night. Predictions from all test nights were then aggregated to assess the overall performance of the modeling approach. Abbreviations: CV: cross-validation

Two outcomes were modeled: (1) next-day migraine and (2) next-day headache (both migraine and non-migraine headache). All features were centered and scaled before model training. Model development followed a nested validation and testing framework designed to prevent bias from using data collected on the same night in both training and evaluation (Fig. 1). For each iteration, one entire night was first excluded (leave-one-night-out; L1NO) as an independent test night and set aside from all model tuning. The remaining nights were then used for model training and validation. During model tuning, a leave-two-nights-out approach (L2NO) approach was applied, in which data from one headache night and one non-headache night were withheld for validation, while remaining nights were used for training (see Supplementary Methods). After tuning, the optimized model was retrained on all non-test nights and evaluated on the held-out test night. After all nights had been tested, nightly predictions were aggregated to obtain overall model performance for each participant. Performance was quantified using sensitivity (recall), specificity, accuracy, precision (positive predictive value), area under the precision-recall curve (AUPRC), true-positive rate for the chosen headache status, and area under the receiver-operating characteristic curve (AUROC).

### Selected Participant Analyses

The highest-performing participant from each individualized model; one for next-day migraine prediction and one for next-day headache prediction, was selected for detailed exploratory analysis. This examination included evaluating varying decision thresholds (the cut-off probability used to classify outcomes) specifically 25%, 50% (default), and 75% to examine how sensitivity, precision, and overall accuracy changed across operating points. SHapley Additive exPlanations (SHAP) analyses to determine which features contributed most strongly to predictions. Features with a SHAP value of ≥ 0.25 or ≤ −0.25 are presented. In exploratory analyses, generalized additive models (GAM) were applied to compare nocturnal dynamics of PRV and EDA, the two autonomic nervous system (ANS)-related metrics identified by ML models as most predictive of headache and migraine. All analyses were conducted in R version 4.4.0 [26].

## RESULTS

### Study Population:

**Figure 2:**
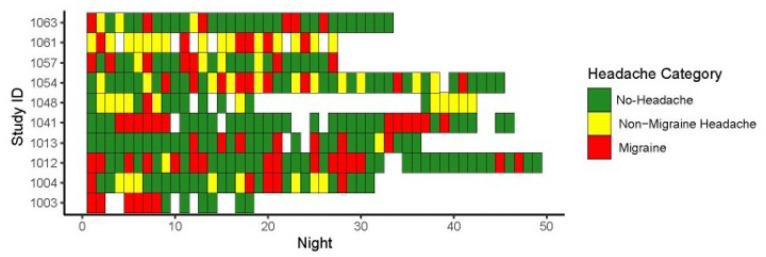
Next-day headache status for each participant. Tile map showing nights on the x-axis and study ID on the y-axis. Each tile is colored according to the participant’s reported headache status on the following day. White spaces indicate days where no watch data was recorded due to technical difficulties or improper usage by participants.

Ten participants (8 female, 2 male) with a median age of 45 (interquartile range (IQR) = 34, 52) were enrolled (Table 1). The median duration of migraine was 25.0 years (IQR = 10, 29). Anxiety was the most common medical co-morbidity (**Supplemental Table 1**) Of these participants, 5 had episodic migraine and 5 had chronic migraine. 7 participants had migraine with aura. (Table 1) A total of 315 nights of data were available for analysis, corresponding to 183 headache-free days, 79 days with migraine, and 54 days with non-migraine headache (Table 1 and Figure2). Participants spent an average (mean ± standard deviation) of 8.5 ±1.8 hours in bed each night (range 3.4–25.0 hours) and slept for 7.5 ±1.4 hours (range 2.9–18.0 hours). Patients wore the device an average of 36.5 nights ± standard deviation of 9.9 days.

### Empatica EmbracePlus Tolerability Data Collection Efficiency:

The EmbracePlus wearable was well tolerated by 8 participants; however, two individuals reported discomfort from the watchband while sleeping. For one participant (1003), the discomfort resulted in discontinuation 10 days prior to planned study termination; for the second, it did not interfere with her ability to finish the study. One participant (1048) ended participation due to repeated technological difficulties with the application synchronizing to the device, which was related to the strength of the cellular network at her residence. Interestingly, six participants requested to wear the watch *longer* than the planned study duration of four weeks, as they reported enjoying learning about their physiology in real time from the Empatica health platform installed on their smartphone. The efficiency, calculated as number of nights with analyzable data/total nights worn, was greater than 75% for 8 of 10 participants with a mean of 86.5% (range: 50% –100%, standard deviation, 17.1%) (Table 1).

### Group level analysis of ANS and sleep features during nights preceding days with no migraine, migraine, and non-migraine headache:

Linear mixed-effects models (LMM) compared physiological measures and sleep metrics between nights prior to no headache, migraine, and non-migraine headache at the group level. EDA reached higher maximums during nights that preceded days with migraine compared to nights that preceded non-migraine or headache free days. The mean maximum EDA of 1.13 uS ± 2.1 uS during nights preceding migraine, was 50.6% higher than nights preceding non-migraine headaches (0.75uS ±1.3 uS). However, global LMM p-values corrected for repeated measures were not significant, underscoring the importance of individualized prediction models.

### Next-Day Migraine Prediction:

Regarding next-day migraine prediction, for 5 of 9 participants, the individualized models demonstrated better than random classification performance by both AUROC and AUPRC (Table 3). Four of 5 participants with episodic migraine had better than random classification of next-day migraine prediction as measured by AUROC and all 4 patients with episodic migraine with aura had better than random classification. The highest AUROC values were observed in participants 1041 and 1054, both of whom had episodic migraine with aura. In contrast, 4 of 5 patients with chronic migraine performed below random classification by both AUROC and AUPRC.

### Next Day Headache Prediction:

7 participants reported more than one non-migraine and migraine headache during the monitoring period and were included in next-day headache models to determine if modeling performance improved when non-migraine and migraine headaches were grouped together. For one participant, this approach significantly improved predictive performance (AUROC increased from 0.68 to 0.81), suggesting that, for this participant, ANS and sleep patterns of headaches reported as non-migraine headaches were similar to those reported as migraine (Table 3). For 2 participants, model performance of next-day headache decreased and for 4 participants, the performance was not significantly changed. (Table 3)

### Selected Participant Analyses:

To explore model behavior in greater detail, SHAP and GAM analyses were performed for the two highest performing models: participant 1041 (next-day migraine) and participant 1054 (next-day headache). These exploratory analyses aimed to identify autonomic and sleep metrics predictive of next day headache and migraine, as well as individuals most likely to benefit from overnight use of the Empatica Embrace device for migraine and headache prediction.

**Figure 3:**
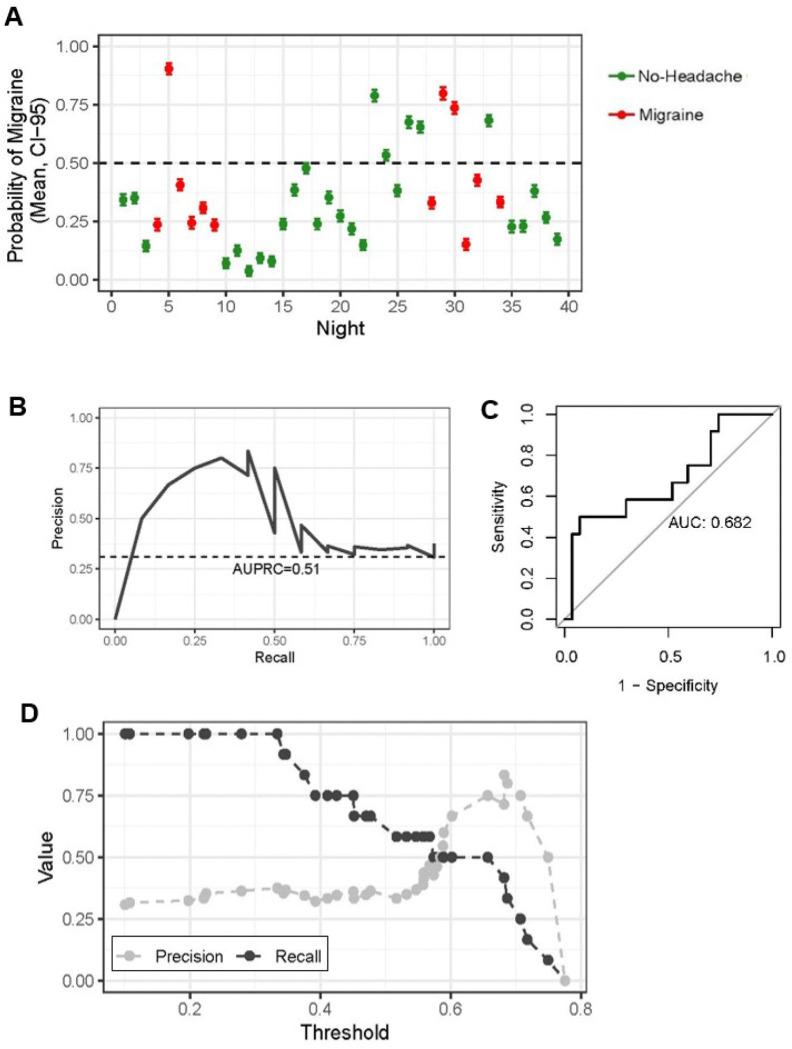
Model performance for participant 1041, highest prediction for next-day migraine. Scatter plot (A) shows mean (+/− 95% CI) predicted probability of headache for each night. The true headache status for each night is indicated by the color. (B) Precision-recall curve for participant 1041, the AUPRC (51%) and outcome prevalence (31%) are indicated. (C) Receiver Operating Characteristic (ROC) curve for participant 1041 migraine vs no-migraine model. (D) Line plot shows precision and recall for participant 1041 at different decision thresholds.

**Figure 5:**
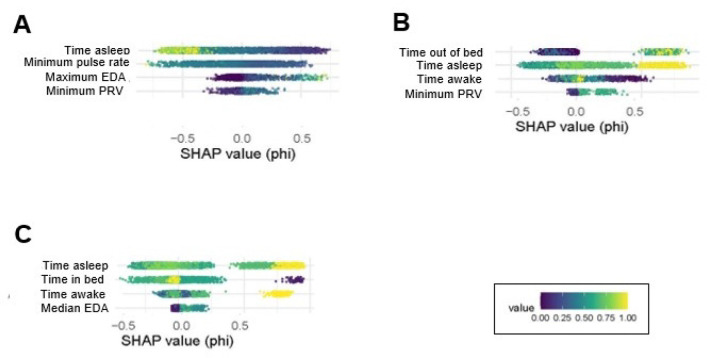
SHAP scores for participants 1054 and 1041. (A) SHAP scores for next-day migraine prediction for participant 1041, (B) SHAP scores for next-day headache prediction for participant 1054, and (C) SHAP scores for next-day migraine prediction for participant 1054. The x-axis (phi) represents the SHAP value, indicating how much each feature (y-axis) changes the predicted probability of next-day headache or migraine when added to the model. Point colors show the normalized feature values (scaled 0–1), with lower values in purple and higher values in yellow, transitioning through blue-green hues. Features with SHAP values ≥ 0.25 and ≤ −0.25 are presented.

*Participant 1041,* a 47-year-old female with episodic migraine with aura and no other medical comorbidities, had the highest performance for next-day migraine prediction. During the study period, 12 days with migraine, 0 days with non-migraine, and 34 days of no headache were reported. A decision threshold of 25% was selected for detailed evaluation because it achieved 100% sensitivity, meaning the model identified all migraine days, though at the expense of low precision of 36%, reflecting a higher rate of false-positive (64%) (**Table 5**, Figure 3). SHAP analyses identified the most influential Empatica features as the time spent asleep, with reduced time asleep predicting an increased likelihood of next-day migraine (Figure 5A). With regards to nocturnal autonomic activity, greater variability in the ANS as measured by lower minimum pulse rate, increased standard deviation (SD) in respiratory rate, and increased SD in pulse rate was predictive of next-day migraine (Figure 5A). A higher maximum EDA was also predictive of next-day migraine (Figure 5A).

### Participant 1054:

Participant 1054 is a 23-year-old female with episodic migraine with aura who reported both non-migraine and migraine headaches and had the highest performance for next-day headache prediction. For this participant, results were relatively consistent across thresholds, but the 50% decision threshold provided a balanced trade-off between sensitivity (75%) and precision (71%), minimizing both false negatives and false positives. At this threshold, the model correctly identified 15 of 20 headache days. For participant 1054, SHAP demonstrated that 3 of the top 4 contributing features to next-day headache prediction were related to sleep. Increased sleep time and interruptions were both associated with next-day headache (Figure 5B). Time spent awake showed no consistent directional trend, indicating a weaker or variable relationship with headache prediction. Similar to 1041, a higher minimum PRV was also linked to greater next-day headache probability. Given 1054 reported both non-migraine and migraine headache days, we next examined next-day migraine prediction. Although sleep features influenced both next-day migraine and next-day headache models, EDA only influenced next-day migraine and *not* next-day headache (Figure 5C).

### Generalized Additive Models (GAM):

**Figure 6:**
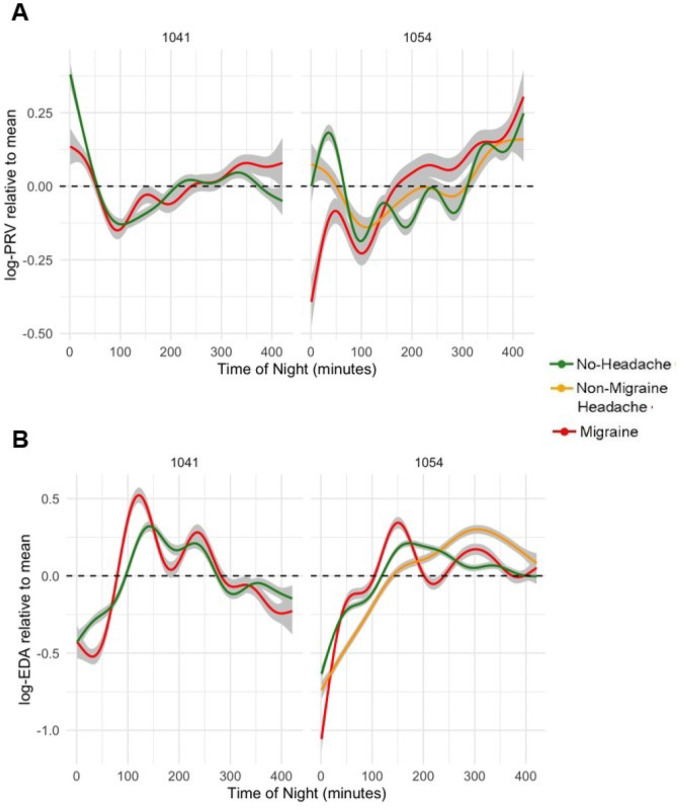
Temporal analysis of PRV, EDA during sleep. Plots show PRV (A) and EDA (B) across the first 420 minutes of sleep for participants 1041 and 1054 using generalized additive models. The gray bands indicate 95% confidence intervals.

For both 1054 and 1041, SHAP analyses identified PRV and EDA as the most contributory autonomic nervous system (ANS) metrics. To further examine their relevance, we analyzed the temporal dynamics of PRV (Figure 6A) and EDA (Figure 6B) over the course of sleep, in relation to next-day migraine, non-migraine headache, and no headache. For both participants 1041 and 1054, PRV showed greater fluctuation during nights preceding migraine; PRV was lower during the initial 100 minutes of sleep and began to increase after ~150 minutes of sleep. PRV remained higher in these later phases of sleep on nights preceding migraine (Figure 6A). Regarding the EDA temporal pattern, we also see greater fluctuations during nights preceding migraine with peaks occurring approximately 120–150 minutes after sleep onset for both participants.

## DISCUSSION

This study aimed to evaluate the feasibility of using wearable-derived autonomic nervous system (ANS) and sleep data to predict migraine and to advance our understanding of how sleep disruption and nocturnal ANS alterations may relate to next-day migraine onset. Person-specific machine learning (ML) models demonstrated better-than-random predictive performance for next-day migraine in 80% of patients with episodic migraine but in none with chronic migraine. While sleep-related features were important for both prediction of next-day migraine and next-day headache, heightened sympathetic nervous system (SNS) activity, as measured by EDA, showed promise as a biomarker to distinguish between nights prior to non-migraine and migraine headaches.

Our study is the first to apply a rigorous L2NO modeling approach to wearable-derived nocturnal ANS and sleep features in patients with chronic and episodic migraine. This work builds on two prior investigations that reported next-day migraine prediction accuracies ranging from 0.60 to 0.90 using wearable-derived nocturnal ANS metrics [7, 27]. However, in those studies, models were trained on short temporal segments (e.g., 5-, 10-, 30-minute windows) rather than aggregated full-night recordings [7, 27]. Aligning with findings by Siirtola and colleagues [27], we found that individualized models showed better performance than group level models. LMMs showed no significant group-level differences between ANS and sleep metrics on nights preceding migraine, non-migraine headache, and headache free day, reflecting the inter-individual variability in migraine prodrome and pathophysiology [13, 27]. However, individualized ML models exhibited better-than-random predictive performance for next-day migraine in 50% of our population, all of whom had episodic migraine, though improvement over chance was modest in most. Reduced accuracy of next-day migraine models in patients with chronic migraine may be due to pathophysiology involving central pain pathways not directly measured by the wearable. Further, patients with chronic migraine frequently experience a shift in neurological baseline [28–30] and thus sleep and ANS activity may be continuously disrupted, reducing the delta between nocturnal activity prior to a migraine and a headache free day. Our findings add to the body of literature demonstrating chronic migraine is *not* just more frequent episodes of migraine; it is a distinct disease.

It is common in clinical practice for patients to report more than one type of headache. However, it is not known whether the pathophysiology of headaches that fail to meet criteria for migraine and those that do meet criteria for migraine have distinct pathophysiology [31]. Although our finding requires replication in larger and diverse patient populations, both group-level LLM and person-specific ML models suggests that EDA, which is driven by a descending sympathetic neural pathway that begins in the hypothalamus, may differentiate nights preceding non-migraine and migraine headaches. This finding has face validity, as the hypothalamus is integral to the premonitory phase of migraine [32, 33]. Interestingly, LLM analyses demonstrated lower average PRV on nights prior to non-migraine nights compared to nights preceding days without headache and days with migraine. PRV is thought to reflect general cardiovagal activity and is modulated by central autonomic networks involving prefrontal-amygdala pathways; resting PRV is decreased in stress-related mood disorders [34]. We believe this pattern should be investigated in future studies, as it suggests average nightly PRV may be more relevant to the forecasting of non-migraine headaches, while SNS activity, as measured by EDA, may be more important for the forecasting of migraine. GAM modeling of highest performing models demonstrated that the early and late stages of sleep may be time periods when ANS activity is most likely to predict next-day migraine and headache; however, this exploratory finding requires further study.

The importance of consistent, sufficient, and uninterrupted sleep for migraine prevention has been well-established [35–37]. Aligning with this body of literature, we found that shorter sleep duration increased the probability of a next-day migraine episode for 1041. In contrast, for participant 1054, *increased* time asleep associated with next-day migraine and headache. These results demonstrate that deviations in sleep, in both the positive and negative direction can increase the likelihood of having a headache align with findings by Stubberud and colleagues that showed increased sleep duration may be protective against migraine for some individuals, but heighten vulnerability to migraine in others [13]. This work underscores the importance of person-specific modeling and supports a growing literature that demonstrates shifts in circadian rhythms may trigger migraine [38, 39]. Alternatively, it is possible that, for some individuals, increased time asleep reflects the premonitory phase of a migraine; imaging studies have demonstrated that alterations in hypothalamic activity, which regulate sleep and circadian rhythm, occur during the premonitory phase of migraine [32, 33].

Interestingly, the highest performing models demonstrated that an increase in *minimum* nocturnal PRV was associated with next-day migraine and headache. While this finding may seem counterintuitive, as higher PRV is generally associated with better overall health and reduced headache [40, 41], there is a normal circadian variation in PRV. PRV is significantly reduced during rapid eye movement (REM) sleep compared to non-REM (NREM) sleep and thus a blunted variation in PRV may indicate the presence of altered sleep architecture that increases vulnerability to next-day migraine [42]. This finding is supported by literature demonstrating that both adults and children with migraine have both decreased amount of REM and altered sleep micro-architecture including a lower index of arousal during REM sleep when compared to patients without migraine [43, 44].

Our study had several limitations. First, although our modest sample size is similar to other studies applying ML modeling to multi-modal high frequency wearable data [7, 24], it prohibited examination of sex-differences. This is an important limitation, given well-established sex differences in ANS regulation and will be addressed in future larger studies [45, 46]. Second, our analyses did not examine EDA spike or storm activity, which have been associated with emotional arousal, reflective of salience network input to the hypothalamus [47]. Future studies will determine if including EDA spike analyses as a model feature improves next-day migraine prediction accuracy. Third, while we found maximum EDA was higher on nights preceding a migraine compared to nights preceding a non-migraine headache; we did not require patients to report how a non-migraine headache failed to meet migraine criteria (i.e., length, severity, or character). Therefore, future planned studies will pair ecological momentary assessments (EMA) with wearable data.

In conclusion, our study demonstrated that personalized ML models incorporating nocturnal high frequency ANS and sleep data from wearable devices have the potential to offer clinical value in predicting migraine attacks. At present, predictive success was modest for most participants and limited to patients with episodic migraine. However, EDA, PRV and sleep features emerged as potentially useful biomarkers of increased migraine vulnerability. Future research should expand nocturnal EDA analyses, exploring whether it may differentiate between non-migraine and migraine headaches in larger populations. While the marked interindividual variability in predictive performance complicates the development of group level “one-size-fits-all” models, this result is important because it highlights the heterogeneity of migraine pathophysiology and underscores the potential for wearables to define mechanistic subgroups of migraine that are not currently captured by clinical assessment. In the future, defining migraine subgroups based on pathophysiology may improve migraine prediction, as well as treatment precision.

## Supplementary Material

Supplementary Files

This is a list of supplementary files associated with this preprint. Click to download.

• SupplementalData.docx

• SupplementalData.docx

## Figures and Tables

**Figure 4:**
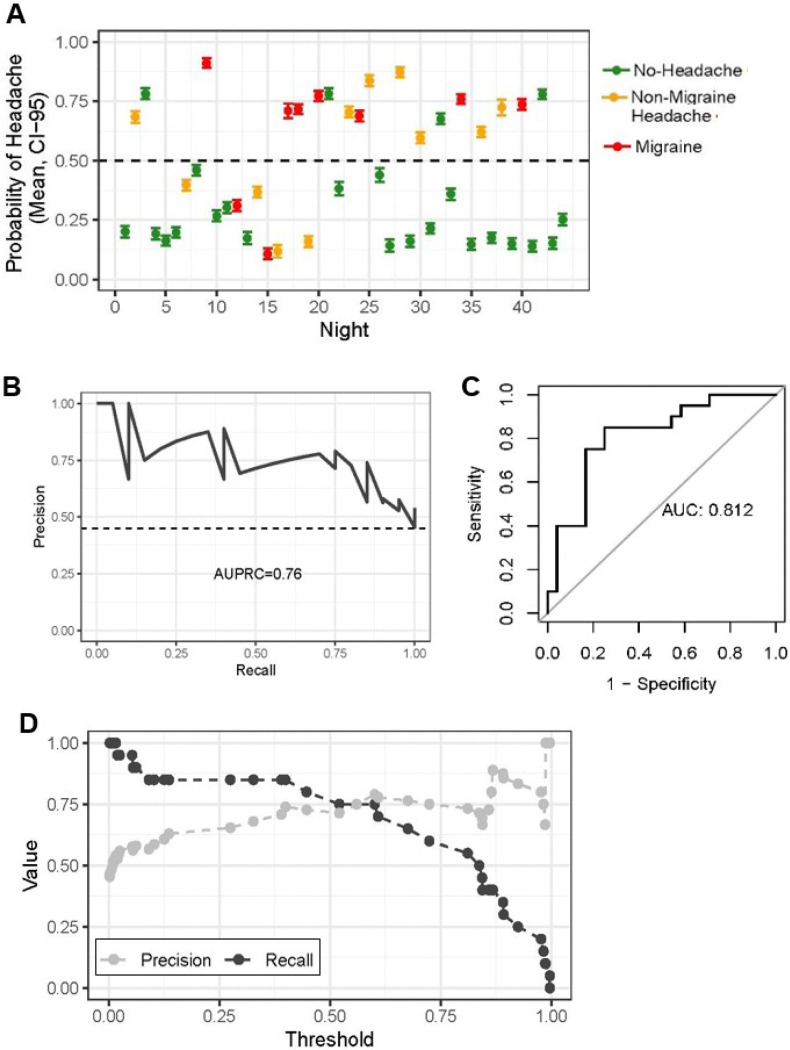
Model performance for participant 1054, highest prediction for next-day headache. (A) Scatter plot of predicted probability (+/− 95% CI) for next-day migraine and non-migraine headache. (B) Precision-recall curve, the AUPRC (76%) and outcome prevalence (45%) are indicated. (C) Receiver Operating Characteristic (ROC) curve for next-day headache. (D) Receiver Operating Characteristic (ROC) curve for next-day headache. Line plot shows precision and recall at different decision thresholds.

**Table 1:** Clinical, demographic, and Empatica Embrace wear-time information for study population

ID	Sex	Age	Migraine Diagnosis		Migraine prevention		Nocturnal data collection time, hours*	Empatica Efficiency^a^
				Years With Migraine		Migraine abortive		

1003	Female	48	Chronic migraine without aura	35	Gabapentin onabotulinumtoxinA Galcanezumab-gnlm	Rizatriptan	8.0 (1.3)	12/18 67%

1004	Female	40	Episodic migraine with aura	25	Atogepant	Rizatriptan	9.1 (1.0)	31/31 100%

1012	Male	25	Episodic migraine without aura	15	None	Advil Ubrogepant	7.8 (2.0)	47/49 96%

1013	Female	43	Chronic migraine with aura	27	Atogepant onabotulinumtoxinA Gabapentin	Rizatriptan Rimegepant	10.3 (1.8)	34/36 94%

1041	Female	47	Episodic migraine with aura	10	Gabapentin Galcanezumab-gnlm	Ubrogepant	7.0 (0.9)	39/47 85%

1048	Female	37	Chronic migraine with aura	25	Gabapentin Galcanezumab-gnlm onabotulinumtoxinA	Rimegepant Advil	7.1 (1.1)	21/42 50%

1054	Female	23	Episodic migraine with aura	8	None	Rimegepant	9.3 (1.5)	44/45 98%

1057	Female	56	Episodic migraine with aura	38	Galcanezumab-gnlm	Advil Ubrogepant	8.4 (0.6)	27/27 100%

1061	Male	57	Chronic migraine without aura	8	Topiramate onabotulinumtoxinA	Ubrogepant Naproxen	9.0 (1.0)	21/27 78%

1063	Female	50	Chronic migraine with aura	27	Topiramate onabotulinumtoxinA	Rimegepant	8.3 (0.4)	33/33 100%

* Data presented is median (IQR)

a calculated as number of nights with analyzable data/total nights worn.

**Table 2:** Nocturnal Wearable Metrics*

	All (N = 315)	Night preceding no headache (N = 183)	Night preceding non-migraine headache (N = 54)	Night preceding Migraine (N = 79)	p-value
Time in bed (minutes)	504.0 [446.8, 563.3]	505.0 [453.0, 566.0]	514.5 [454.5, 560.5]	499.0 [439.0, 558.5]	0.591
Time sleeping (minutes)	443.0 [397.8, 498.0]	443.0 [398.0, 495.5]	464.0 [408.8, 500.5]	434.0 [390.5, 498.0]	0.488
Time awake in bed (minutes)	53.5 [39.0, 72.3]	54.0 [40.5, 73.0]	46.0 [35.3, 67.5]	54.0 [39.0, 72.0]	0.411
Electrodermal activity (EDA) microsiemens (μS)	0.3 [0.1, 1.0]	0.3 [0.1, 0.8]	0.4 [0.2, 0.8]	0.4 [0.2, 1.3]	0.102
Pulse rate (beats per minute)	69.8 [65.9, 75.7]	70.4 [66.7, 76.0]	67.3 [64.4, 72.7]	70.6 [65.7, 76.9]	0.210
Temperature C°	34.5 [33.9, 34.9]	34.5 [33.9, 34.9]	34.5 [34.0, 34.8]	34.4 [33.8, 35.7]	0.110
Pulse rate variability (PRV)	26.7 [17.7, 34.1]	26.7 [18.1, 33.5]	24.2 [18.2, 34.4]	27.1 [16.5, 35.1]	0.533
Respiratory Rate (breaths per minute)	16.4 [15.7, 17.3]	16.4 [15.5, 17.4]	16.1 [15.4, 16.7]	16.4 [16.0, 18.2]	0.501

* Data is presented as median [interquartile range]

**Table 3:** Next day migraine and next day non-migraine model performance.

	Migraine vs No-Migraine					Headache vs No-Headache		
ID	Model	AUPRC	Prevalence	AUROC	Model	AUPRC	Prevalence	AUROC
1003	Elastic-Net	62.10%	50%	52.80%	-	-	-	-
1004	Elastic-Net	18.80%	16.10%	57.70%	GBM	39.30%	41.90%	46.20%
1012	GBM	30.30%	34.00%	43.80%	GBM	27.60%	36.20%	34.90%
1013	RF	15.60%	20.60%	37.00%	RF	19.20%	23.50%	40.40%
1041	Elastic-Net	51.20%	30.80%	68.20%	-	-	-	-
1048	-	-	-	-	GBM	61.40%	57.10%	61.10%
1054	GBM	37.20%	20.50%	67.90%	RF	76%	45.50%	81.20%
1057	GBM	26%	25.90%	52.90%	Elastic-Net	43.60%	40.70%	56.80%
1061	RF	27.20%	28.60%	49.40%	-	-	-	-
1063	RF	12.70%	18.20%	31.50%	Elastic-Net	18.90%	27.30%	28.20%

Abbreviations: AUPRC, Area Under the Precision-Recall Curve; AUROC, Area Under the Receiver-Operating Curve; GBM, Gradient Boosting Machines; RF, Random Forest.

**Table 4:** Threshold-specific metrics for participants 1054 and 1041.

ID	Model	Outcome	Threshold	Precision	Accuracy	Sensitivity	Specificity	Headache/Migraine Days*	Total Days
1054	Random Forest	Headache	25%	65.4%	72.7%	85.0%	62.5%	20	44
1054	Random Forest	Headache	50%	71.4%	75%	75.0%	75.0%	20	44
1054	Random Forest	Headache	75%	73.3%	70.5%	55.0%	83.3%	20	44
1041	Elastic-Net	Migraine	25%	36.4%	46.2%	100.0%	22.2%	12	39
1041	Elastic-Net	Migraine	50%	33.3%	51.3%	58.3%	48.1%	12	39
1041	Elastic-Net	Migraine	75%	0%	66.7%	0%	96.3%	12	39

* For participant 1054, headache days were modeled and for participant 1041, migraine days were modeled.

## Abbreviations

ML: Machine Learning
EDA: Electrodermal Activity
PR: Pulse Rate
PRV: Pulse Rate Variability
RR: Respiratory Rate
AUROC: Area Under the Receiver-Operating Curve
AUPRC: Area Under the Precision-Recall Curve
SHAP Analysis: SHapley Additive exPlanation Analysis
GAM: Generalized Additive Model
ANS: Autonomic Nervous System
SNS: Sympathetic Nervous System
MICE Framework: Multivariate Imputation by Chained Equations Framework
L1NO: Leave-One-Night Out
L2NO: Leave-Two-Nights-Out
LMM: Linear Mixed-Effects Models
LLM: Large Language Model
